# Incidence study of precocious puberty in the population of the state of Goiás: did the COVID-19 pandemic modify the epidemiology?^✰^

**DOI:** 10.1016/j.jped.2026.101590

**Published:** 2026-07-29

**Authors:** Renata Machado Pinto, Gabriela Luz Castelo Branco de Souza, Gustavo Moraes Magalhães, Isabely Gelinski, Luiz Felipe Macedo Silva

**Affiliations:** a Universidade Federal de Goiás (UFG), Faculdade de Medicina, Department of Pediatrics and Childcare, Goiânia, GO**,** Brazil; b Universidade Federal de Goiás (UFG), Faculdade de Medicina, Goiânia, GO**,** Brazil

**Keywords:** Central precocious puberty, COVID-19, Lockdown, Puberty, Gonadotropin-releasing hormone agonists

## Abstract

**Objective:**

To evaluate the incidence and epidemiological, clinical, and laboratory characteristics of Central Precocious Puberty (CPP) before, during, and after the COVID-19 pandemic in Goiás, Brazil.

**Method:**

This retrospective, longitudinal, observational study with a quantitative approach was conducted in Goiás using administrative records of high-cost medication dispensing. All patients registered in the state dispensing center who initiated treatment between 2018 and 2023 were included. Data were analyzed using the Statistical Package for Social Sciences (IBM Corporation, Armonk, USA), version 26.0. Statistical significance was set at 5% (p < 0.05).

**Results:**

A total of 910 CPP treatment requests were evaluated, including 153 before the pandemic, 390 during 2020–2021, and 367 during 2022–2023. This corresponded to increases of 154% and 140% during the pandemic and post-pandemic periods, respectively (p < 0.001). From 2020 onward, patients started gonadotropin-releasing hormone analog therapy at younger ages (104.84 ± 12.52 vs. 107.17 ± 13.42 months; p = 0.027), but showed more advanced pubertal development, with higher Tanner stages (B/G stage 4: 15.9% and 21.4% vs. 8.6%; p = 0.035) and higher stimulated LH levels (22.39 ± 16.81 and 24.64 ± 22.13 vs. 19.87 ± 18.08 mIU/mL; p = 0.046).

**Conclusions:**

CPP incidence increased significantly in Goiás during and after the COVID-19 pandemic. Patients also presented more advanced clinical and laboratory features than those diagnosed before the pandemic.

## Introduction

Puberty is the process that marks the transition from childhood to adulthood. It is characterized by the gradual activation of the hypothalamic-pituitary-gonadal (HPG) axis and the development of secondary sexual characteristics, culminating in reproductive maturity[1,2]. Precocious puberty (PP) is defined by the development of secondary sexual characteristics before 8 years of age in girls and 9 years of age in boys. Early puberty, however, refers to pubertal onset occurring earlier than the population average but still within the normal age range; despite sharing certain clinical manifestations with PP, it represents a distinct condition and should not be considered synonymous with precocious puberty[3]. It can be classified as central, characterized by premature activation of the HPG axis, or peripheral, resulting from autonomous production of sex steroids independent of HPG axis activation[4].

Central precocious puberty (CPP) is the most common form and affects girls 10 to 23 times more often than boys[3]. In girls, precocious puberty is most often idiopathic. Furthermore, early puberty, which occurs more frequently and is more readily recognized in females, may increase referral rates for endocrinological evaluation, thereby contributing to the greater detection of precocious puberty in this population. In boys, it is frequently linked to neurological alterations, such as hamartomas and tumors of the central nervous system[2,3]. Proper diagnosis and management are fundamental, as early exposure to sex steroids leads to accelerated growth, premature epiphyseal fusion, and consequent compromise of final height, in addition to possible psychosocial repercussions[3,4].

Several centers have reported a rise in suspected cases of CPP since the onset of the COVID-19 pandemic. However, it is crucial to specify that this surge refers strictly to idiopathic forms of central precocious puberty, whereas organic forms have remained stable in incidence even during the COVID-19 period[5]. While organic CPP is strongly predicted by independent factors such as a younger age at diagnosis, accelerated growth velocity, and a peak luteinizing hormone (LH) level > 9.1 mUI/mL[5], the rise in idiopathic cases is primarily linked to lifestyle modifications. Studies suggest that factors such as a sedentary lifestyle, increased screen exposure, sleep disturbances, dietary changes, and psychological stress may have contributed to this increase in idiopathic CPP[6,7]. However, national and regional data on CPP epidemiology remain scarce, particularly regarding temporal trends before and after the COVID-19 pandemic. This knowledge gap highlights the need for local population-based studies to better characterize disease patterns and support public health planning and decision-making.

Therefore, this study aims to evaluate the incidence of CPP in Goiás, Brazil, and compare the clinical and laboratory characteristics of affected patients before, during, and after the COVID-19 pandemic. Specifically, it sought to characterize temporal trends in CPP from 2018 to 2023, compare clinical and laboratory characteristics of patients across different periods, and investigate whether the pandemic period was associated with changes in disease presentation and severity.

## Methods

This retrospective longitudinal observational study was conducted in Goiânia, Goiás, Brazil, based on analysis of data obtained from records archived at Juarez Barbosa State Center for High-Cost Medication (CMAC-JB), the state reference center responsible for the dispensing of gonadotropin-releasing hormone analogs (GnRHa).

The study population consisted of children diagnosed with Central Precocious Puberty (CPP) registered at CMAC-JB for GnRHa dispensing, with treatment initiation documented between January 2018 and December 2023. The following variables were collected: sex, age at GnRHa request, age at onset of pubertal symptoms, comorbidities, bone age, Tanner staging, weight, height, body mass index (BMI), basal and stimulated LH levels; for girls, the occurrence of menarche, basal and stimulated estradiol, and ultrasound determination of uterine and ovarian volumes were also evaluated, and for boys, basal testosterone.

For diagnostic confirmation of CPP, basal and stimulated LH levels were evaluated in accordance with current diagnostic recommendations. Basal LH values > 0.3 IU/L by immunochemiluminometric assay (ICMA) or > 0.6 IU/L by immunofluorometric assay (IFMA) were considered suggestive of CPP, although low basal gonadotropin levels did not exclude the diagnosis[8]. A stimulated LH peak >5 IU/L after GnRH stimulation testing was used as the cutoff for hypothalamic-pituitary-gonadal axis activation[9]. In cases evaluated with leuprolide stimulation testing, pubertal response was defined as stimulated LH > 10 gt; 10 IU/L by IFMA or > 8 gt; 8 IU/L by chemiluminescence/electrochemiluminescence assays[8]. Pelvic ultrasonography with evaluation of uterine and ovarian volumes was also used as a complementary diagnostic tool in girls.

Patients were included in the study if they met all of the following criteria: active registration at CMAC-JB; initiation of GnRHa treatment between 2018 and 2023; diagnosis of central precocious puberty identified by ICD-10 codes E30.1 or E22.8; availability of complete data regarding age at referral, age at onset of pubertal symptoms, bone age, Tanner pubertal staging, weight, height, and basal and stimulated LH levels. Patients with records unavailable in the archive system, with incomplete or inconsistent information, and patients with a diagnosis other than CPP were excluded. According to these criteria, only 910 patient records were eligible for inclusion. This selection process may have introduced selection bias and bias arising from non-random missing data, as patients who underwent more comprehensive diagnostic evaluation were more likely to be included. Consequently, the final sample may not be fully representative of the population served by the institution. Nevertheless, complete-case analysis was required to ensure the methodological consistency, validity, and reliability of the study data. Some patients were older than the classical age thresholds for CPP at the time of treatment request. However, they were included because the diagnosis was based on documented pubertal onset before 8 years in girls and 9 years in boys, regardless of age at referral or treatment initiation.

Data were collected using a standardized form developed by the researchers that included all variables of interest for the study. Both paper and electronic records related to GnRHa dispensing and follow-up at CMAC-JB were analyzed, including registration forms, medical reports, medication requests, growth charts based on World Health Organization (WHO) standards, and hand and wrist radiography reports. Data were systematically extracted in accordance with ethical and confidentiality standards and subsequently organized into an electronic database for statistical analysis.

Data were stratified by sex and year of treatment initiation. Patients were categorized into three time periods according to the year of GnRHa request at CMAC-JB: Pre-Pandemic (2018–2019), Pandemic (2020–2021), and Post-Pandemic (2022–2023). This division was based on the period of COVID-19-related lockdown and social restriction measures in Brazil, aiming to evaluate possible temporal changes in CPP incidence and clinical presentation associated with the pandemic context. The annual incidence rates of CPP were calculated by dividing the number of new treatment requests (numerator) by the pediatric population up to 10 years of age in the state of Goiás (denominator). This specific age grouping for the denominator was determined solely by the availability of demographic data from the Brazilian Institute of Geography and Statistics (IBGE) census, which provides population estimates in fixed age blocks. Anthropometric data were evaluated by calculating Z-scores using the AnthroPlus software, developed by the World Health Organization. Pubertal characteristics were assessed using Tanner classification. Bone age was assessed from hand and wrist radiographic reports, and other clinical aspects were evaluated based on the available medical reports. Laboratory aspects were analyzed through medical reports and exams attached to the files of each child requiring medication dispensing for precocious puberty control.

The characterization of the demographic and anthropometric profile, age of incidence, and the impact of the pandemic on these factors was performed using means, standard deviations, absolute and relative frequencies. To assess data normality, a normalized Q-Q plot and a histogram of standardized residuals were used, according to the method proposed by Chambers et al.[9]. An exploratory analysis followed, in which variables were compared between the study years using Pearson's Chi-square and Post hoc Chi-square tests[10]. For continuous variables, Analysis of Variance (ANOVA) followed by Tukey's test was adopted. The comparison of precocious puberty incidence over the years was made using the One-sample Chi-square test. Data were analyzed using the Statistical Package for Social Sciences (IBM Corporation, Armonk, USA), version 26.0. The significance level adopted was 5% (p < 0.05).

The study was approved by the Leyde das Neves Research Ethics Committee, linked to the Faculty of Medicine of the Federal University of Goiás, under Certificate of Presentation for Ethical Consideration (CAAE) No 69,029,923.6.3001.5082 in compliance with the ethical principles of Resolution No 466/2012 of the National Health Council.

## Results

Between 2018 and 2023, 1601 records related to GnRH analog dispensing were identified at CMAC-JB. Of these, 910 patients met the inclusion criteria and were included in the study. Most patients were female (97.5%), with a mean age at GnRHa request of 106.17 ± 12.84 months and a mean age at pubertal onset of 87.27 ± 12.38 months. The demographic and anthropometric characteristics of the study population are summarized in Table 1.

**Table 1:** Characterization of patients with CPP treated at CMAC-JB from 2018 to 2023.

Evaluated Characteristic	
Sex	n (%)
Female	887 (97.5)
Male	23 (2.5)
Age at GnRHa request	n (%)
0 to 59 months	3 (0.3)
60 to 119 months	807 (88.7)
120 months or more	100 (11.0)
Age at GnRHa request - months (Mean ± SD)	106.17 ± 12.84
Age at pubertal onset	n (%)
0 to 59 months	9 (1.1)
60 to 119 months	807 (98.3)
120 months or more	5 (0.6)
Age at pubertal onset - months (Mean ± SD)	87.27 ± 12.38
Weight - kg (Mean ± SD)	34.71 ± 8.25
Height - meters (Mean ± SD)	1.40 ± 0.82
BMI (Mean ± SD)	18.31 ± 3.24
BMI Z-score (Mean ± SD)	0.76 ± 1.27
Height Z-score (Mean ± SD)	1.16 ± 6.25

n, absolute frequency; %, relative frequency; SD, standard deviation.

**Figure 1:**
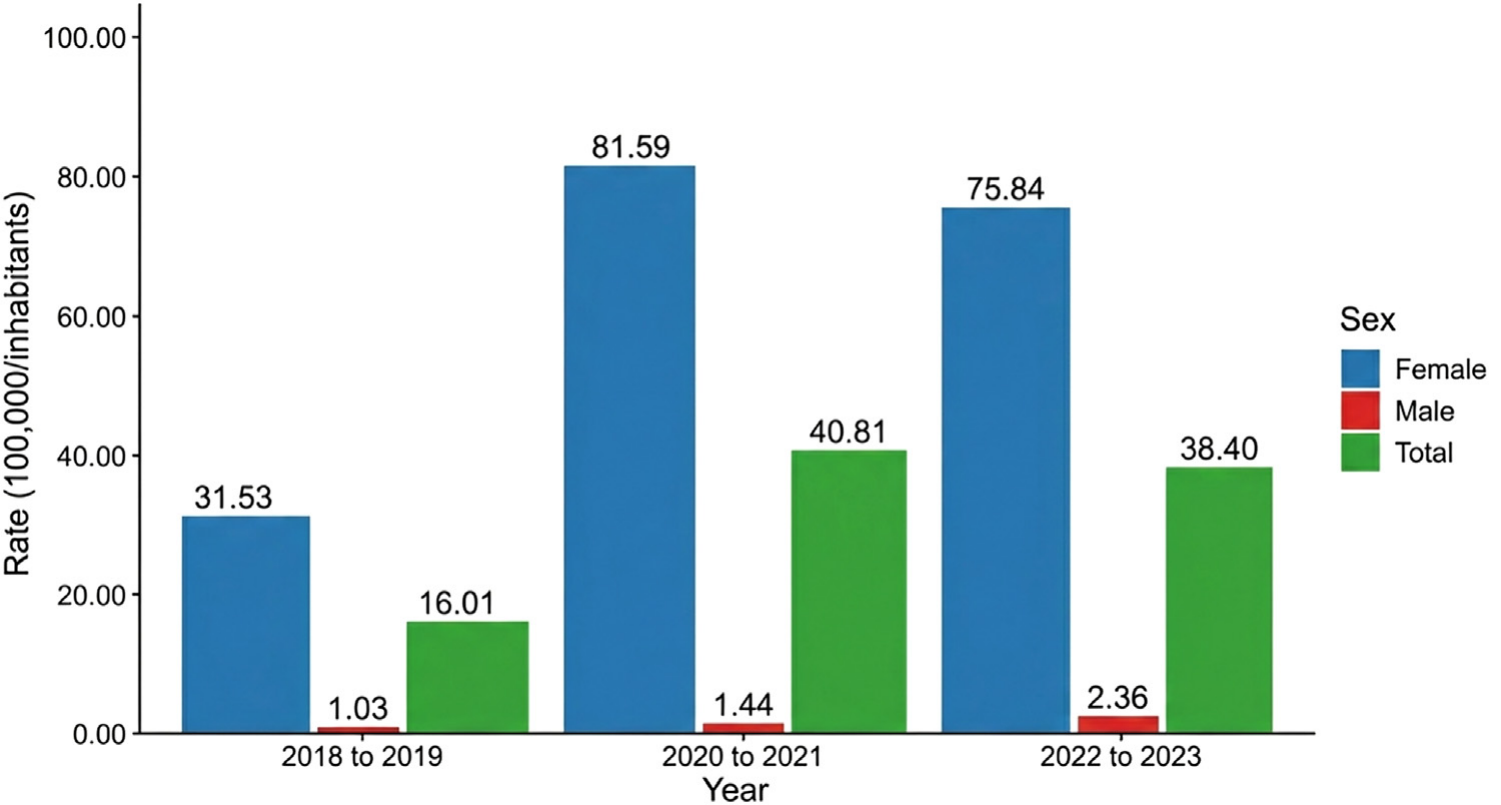
Incidence of CPP per 100.000 inhabitants in Goiás. Stratified by sex in the Pre-Pandemic, Pandemicand Post-Pandemic periods.

In the two-year period preceding the COVID-19 pandemic, 153 children initiated GnRHa treatment; this number increased by 154% during the pandemic, to 390 CPP cases, and remained elevated in 2022–2023, with 367 patients. The incidence of CPP was calculated for the state of Goiás during the Pre-Pandemic, Pandemic, and Post-Pandemic periods, using 2022 census data on the population of children under 10 years old in the state. The overall incidence rose from 16.01 cases per 100,000 children before the pandemic to 40.81 during the pandemic and remained high at 38.40 cases per 100,000 children in the post-pandemic period. Figure 1 presents the incidence per 100,000 inhabitants in each of these analyzed periods.

Comparisons across study periods revealed significant differences between pubertal and hormonal characteristics, as detailed in Table 2, Table 3. Children evaluated during and after the pandemic initiated GnRHa treatment at younger ages than those evaluated before the pandemic (104.84 ± 12.52 and 107.15 ± 12.85 vs. 107.17 ± 13.42 months; p = 0.026). Post-hoc analysis confirmed that this difference was driven primarily by the contrast between the pandemic and post-pandemic periods (p = 0.036 and p = 0.005, respectively), while no significant difference was observed between the pre-pandemic and pandemic periods alone (p = 0.138). Furthermore, pubertal development was more advanced, despite the younger age at treatment initiation, as demonstrated by a higher frequency of Tanner stage B/G 4 during the pandemic and post-pandemic periods compared with the pre-pandemic period (15.9% and 21.4% vs 8.6%, p = 0.035).

**Table 2:** Clinical and laboratory characterization of patients with CPP treated at CMAC-JB according to the period (Pre-Pandemic, Pandemic, and Post-Pandemic).

	Period			p
	Pre-Pandemic (n = 153)	Pandemic (n = 390)	Post-Pandemic (n = 367)	
Sex n (%)				
Female	148 (96.7)	383 (98.2)	356 (97.0)	0.468*
Male	5 (3.3)	7 (1.8)	11 (3.0)	
Age at GnRHa request n (%)				
0 to 59 months	0 (0.0)	2 (0.5)	1 (0.3)	
60 to 119 months	132 (86.3)	358 (91.8)	317 (86.4)	
120 or more	21 (13.7)	30 (7.7)	49 (13.4)	
Age at GnRHa request - months (Mean ± SD)	107.17 ± 13.42	104.84 ± 12.52	107.15 ± 12.85	0.027**
Age at pubertal onset n (%)				
0 to 59 months	2 (1.5)	3 (0.8)	4 (1.2)	
60 to 119 months	130 (96.3)	354 (99.2)	323 (98.2)	
120 or more	3 (2.2)	0 (0.0)	2 (0.6)	
Anthropometric data				
Weight - kg (Mean ± SD)	35.06 ± 7.54	33.86 ± 8.07	35.44 ± 8.64	0.028**
Height - meters (Mean ± SD)	1.37 ± 0.09	1.36 ± 0.08	1.45 ± 1.29	0.313**
BMI (Mean ± SD)	18.43 ± 2.80	18.13 ± 3.23	18.46 ± 3.40	0.331**
BMI Z-score (Mean ± SD)	0.86 ± 1.14	0.73 ± 1.22	0.75 ± 1.38	0.561**
Height Z-score (Mean ± SD)	0.92 ± 1.37	0.80 ± 1.16	1.65 ± 9.72	0.160**
Age at pubertal onset - months (Mean ± SD)	87.01 ± 12.25	86.79 ± 12.53	87.89 ± 12.29	0.491**
Tanner Stage - P n (%)				
1	24 (17.1)	55 (16.5)	42 (12.5)	0.005*
2	49 (35.0)	101 (30.3)	84 (25.0)	
3	60 (42.9)	145 (43,5)	155 (46.1)	
4	5 (3.6)	30 (9.0)	52 (15.5)≠	
5	2 (1.4)	2 (0.6)	3 (0.9)	
Tanner Stage - B/G n (%)				
1	1 (0.7)	3 (0.9)	3 (0.9)	0.035*
2	34 (24.3)	71 (21.3)	53 (15.8)	
3	91 (65.0)	205 (61.6)	206 (61.3)	
4	12 (8.6)	53 (15.9)	72 (21.4)≠	
5	2 (1.4)	1 (0.3)	2 (0.6)	
Occurrence of menarche n (%)				
No	138 (95.8)	354 (94.7)	322 (93.3)	0.512*
Yes	6 (4.2)	20 (5.3)	23 (6.7)	
Bone age (months)				
0 to 59	0 (0.0)	1 (0.3)	1 (0.3)	0.086*
120 to 168	120 (78.4)	302 (77.4)	312 (85.0)	
60 to 118	33 (21.6)	87 (22.3)	54 (14.7)	
	Mean ± SD			
Bone age - months	126.52 ± 16.68	125.14 ± 15.59	128.25 ± 15.47	0.026**
Basal LH - mIU/mL	2.03 ± 2.06	2.50 ± 3.81	2.40 ± 2.74	0.321**
Stimulated LH - mIU/mL	19.87 ± 18.08	22.39 ± 16.81	24.64 ± 22.13	0.046**
Basal E2 - pg/mL	32.55 ± 19.35	38.61 ± 40.98	40.10 ± 39.27	0.671**
Stimulated E2 - pg/mL	30.84 ± 13.08	55.90 ± 0.00	37.00 ± 5.66	0.270**
Uterine volume - cm³	7.44 ± 6.19	7.58 ± 5.17	8.83 ± 6.77	0.167**
Right ovary volume - cm³	2.83 ± 1.94	2.87 ± 1.81	2.93 ± 1.92	0.961**
Left ovary volume - cm³	2.91 ± 2.34	3.00 ± 2.84	2.93 ± 2.60	0.941**
Basal testosterone - ng/dL	59.60 ± 0.85	14.66 ± 9.14	18.55 ± 17.14	0.004**

Legend: * Chi-square; ** ANOVA; ≠ Post Hoc; n, absolute frequency; %, relative frequency; SD, standard deviation.

The mean difference is significant at the < 0.05 level.

**Table 3:** Multiple comparisons of Table 2 according to Tukey HSD.

Dependent Variable			p
Weight (kg)	2018 to 2019	2020 to 2021	0.284
		2022 to 2023	0.882
	2020 to 2021	2018 to 2019	0.284
		2022 to 2023	0.025
	2022 to 2023	2018 to 2019	0.882
		2020 to 2021	0.025
Age at GnRHa request (months)	2018 to 2019	2020 to 2021	0.138
		2022 to 2023	1.000
	2020 to 2021	2018 to 2019	0.138
		2022 to 2023	0.036
	2022 to 2023	2018 to 2019	1.000
		2020 to 2021	0.036
		2022 to 2023	0.005
Bone age (months)	2018 to 2019	2020 to 2021	0.627
		2022 to 2023	0.489
	2020 to 2021	2018 to 2019	0.627
		2022 to 2023	0.019
	2022 to 2023	2018 to 2019	0.489
		2020 to 2021	0.019
Stimulated LH (mIU/mL)	2018 to 2019	2020 to 2021	0.431
		2022 to 2023	0.051
	2020 to 2021	2018 to 2019	0.431
		2022 to 2023	0.268
	2022 to 2023	2018 to 2019	0.051
		2020 to 2021	0.268
Basal testosterone	2018 to 2019	2020 to 2021	0.004
		2022 to 2023	0.005
	2020 to 2021	2018 to 2019	0.004
		2022 to 2023	0.858
	2022 to 2023	2018 to 2019	0.005
		2020 to 2021	0.858

*The mean difference is significant at the < 0.05 level.

Stimulated LH levels were significantly higher during the pandemic and post-pandemic periods than before the pandemic (22.39 ± 16.81 and 24.64 ± 22.13 vs. 19.87 ± 18.08 mIU/mL; p = 0.046). However, post-hoc comparisons revealed that statistical significance was driven primarily by the trend between the pre-pandemic and post-pandemic groups (p = 0.051), without a significant difference between the pandemic and post-pandemic periods (p = 0.268). Table 3 compares the clinical and laboratory profiles of patients according to the period of GnRHa request at CMAC-JB.

## Discussion

The present study demonstrated a significant increase in the incidence of CPP in the state of Goiás during the COVID-19 pandemic, with a 154% increase compared with the pre-pandemic period, and incidence remaining elevated during the post-pandemic period. These findings corroborate data from studies conducted in different countries, including Italy, South Korea, China, Brazil, Turkey, India, and the United States, which reported a significant increase in new CPP cases following the onset of social distancing measures[11–13].

The female predominance observed across all analyzed periods is consistent with the literature, which reports a higher frequency of CPP in girls[12]. However, unlike most published studies, which excluded male patients, the present study also identified an increase in incidence in boys, a finding described in only a few international studies[14]. This finding distinguishes the present study from most previous reports and reinforces the need for the systematic inclusion of males in epidemiological analyses of CPP.

A reduction in the mean age of treatment request was observed during the pandemic period, paradoxically associated with more advanced pubertal stages and higher stimulated LH values. Similar results were described by Stagi et al.[11]. and Goggi et al.[15], suggesting that although the diagnosis occurred chronologically earlier, pubertal progression may have been faster during this period. On the other hand, no significant differences were observed in bone age, BMI Z-score, or uterine and ovarian volumes, which contrasted with some studies that reported greater global maturational advancement during the lockdown[16].

Possible explanations for the observed increase in incidence and CPP progression after 2020 include indirect factors related to lifestyle changes imposed by the lockdown—such as increased screen time, sedentary behavior, sleep disturbances, psychological stress, and dietary changes—as well as a possible direct effect of SARS-CoV-2 infection on the neuroendocrine mechanisms involved in pubertal onset[12]. In Brazil, social distancing policies were implemented through a decentralized approach, with state and municipal governments responsible for establishing and enforcing public health restrictions. In Goiás, mitigation strategies were primarily guided by intensive care unit (ICU) occupancy rates and epidemiological projections developed by the Federal University of Goiás (UFG). Initial measures included the closure of non-essential businesses and educational institutions (March–April 2020), followed by a gradual reopening process. Between June and September 2020, an intermittent quarantine strategy was adopted, consisting of alternating 14-day periods of restriction and reopening. During subsequent pandemic surges in 2021, additional containment measures were implemented and later progressively relaxed as vaccination coverage increased. Throughout the study period, public health interventions were continuously adjusted based on epidemiological surveillance indicators[17,18]. Experimental and clinical evidence suggests that alterations in circadian rhythm and melatonin secretion may modulate this process, although the exact mechanisms remain unclear[19,20].

Regarding nutritional status, no clinically relevant difference in BMI or BMI Z-score was observed between periods, a finding consistent with studies questioning the isolated role of adiposity as a determinant of CPP[16,21]. These results contrast with findings from Asian populations, where elevated BMI was identified as an independent risk factor for CPP, suggesting possible ethnic, cultural, and environmental influences[14].

Hormonal analysis showed stability in basal LH and estradiol levels, whereas stimulated LH increased progressively over the study period, indicating greater gonadotropic activity after the pandemic. Similar results were reported in Italian studies, which also described an acceleration of pubertal progression after the lockdown[11,16], although other studies did not identify significant hormonal alterations, reflecting methodological and population heterogeneity among studies[21,22].

Pelvic ultrasonography is an important adjunctive tool in CPP evaluation, allowing noninvasive assessment of uterine and ovarian development. Recent studies have demonstrated high concordance between pelvic ultrasound findings and the GnRH stimulation test, supporting its usefulness in diagnosing CPP and screening[23]. In the present study, uterine and ovarian volumes remained stable across the analyzed periods, despite increased CPP incidence and more advanced pubertal presentation.

A relevant finding was the reduction in basal testosterone levels in male patients during the pandemic and post-pandemic periods, suggesting diagnosis in less advanced phases of puberty. This result may be related to greater parental observation during the period of social isolation, since the increase in testicular volume is less evident than breast development in girls. The inclusion of male patients, often excluded from similar studies, enabled this analysis and constitutes a key difference of the present study.

Among the limitations, the retrospective design, reliance on secondary data, and the exclusion of a substantial number of records due to incomplete information stand out. The inclusion of some patients older than the classical age thresholds at the time of treatment request reflects real-world delays in referral, diagnostic investigation, and access to specialized care. Although this may introduce selection bias, excluding these patients could underestimate the true burden of CPP in the studied population and reduce the external validity of the findings. Furthermore, it was not possible to directly evaluate behavioral and environmental factors, such as screen time, dietary pattern, physical activity level, family pubertal history, or prior SARS-CoV-2 infection, which could contribute to a better understanding of the mechanisms involved.

The authors acknowledge that analyzing children aged 10 years or less as a single group for incidence calculations is a methodological limitation, as pubertal onset after 8 years in girls is considered physiological. This choice was mandated by the rigid age intervals provided by the IBGE census data. Theoretically, a broader denominator could underreport the true incidence. However, because the CMAC-JB protocol strictly limits GnRHa dispensing to patients with pathological pubertal onset (before 8 years in girls and 9 years in boys), the numerator consists exclusively of true clinical cases. Therefore, this demographic constraint likely led to a more conservative estimate of the incidence rates rather than an overestimation, thereby preserving the validity of the observed epidemiological trends.

In conclusion, this study demonstrates a significant and sustained increase in the incidence of central precocious puberty in the state of Goiás during and after the COVID-19 pandemic, associated with signs of accelerated pubertal progression. The findings reinforce the need for continuous epidemiological surveillance, early diagnosis strategies, and the development of regional protocols adapted to the new post-pandemic reality, as well as for stimulating prospective research that explores, in an integrated manner, the environmental, behavioral, and biological factors involved in CPP.

## Authors’ contributions

Renata Machado Pinto: General research supervision: conceptualization of the research problem, definition of research methodology, data analysis and interpretation, critical review of the manuscript.

Gabriela Luz Castelo Branco de Souza: Data collection, data interpretation, writing of methodology and results, general review of the article.

Gustavo Moraes Magalhães: Data collection, data interpretation, writing of results interpretation, general review of the article.

Isabely Gelinski: Data collection, data interpretation, writing of results discussion, general review of the article.

Luiz Felipe Macedo Silva: Data collection, data interpretation, writing of introduction and methodology, general review of the article.

## Funding source

Nothing to declare.

## Conflicts of interest

The authors declare no conflicts of interest.

## Data Availability

The data that support the findings of this study are available from the corresponding author.
